# Dynamic Arterial Elastance as a Predictor of Supine-to-Prone Hypotension (SuProne Study): An Observational Study

**DOI:** 10.3390/medicina59122049

**Published:** 2023-11-21

**Authors:** Jin Hee Ahn, Jiyeon Park, Jae-Geum Shim, Sung Hyun Lee, Kyoung-Ho Ryu, Taeho Jeong, Eun-Ah Cho

**Affiliations:** Department of Anesthesiology and Pain Medicine, Kangbuk Samsung Hospital, Sungkyunkwan University School of Medicine, Seoul 03181, Republic of Korea; blatt.ahn@samsung.com (J.H.A.); j27.park@samsung.com (J.P.); jaegeum.shim@samsung.com (J.-G.S.); hoho4321.lee@samsung.com (S.H.L.); kyoungho.ryu@samsung.com (K.-H.R.); taeho91.jeong@samsung.com (T.J.)

**Keywords:** dynamic arterial elastance, prone, hypotension, supine-to-prone hypotension, predictor

## Abstract

*Background and Objectives*: Supine-to-prone hypotension is caused by increased intrathoracic pressure and decreased venous return in the prone position. Dynamic arterial elastance (Ea_dyn_) indicates fluid responsiveness and can be used to predict hypotension. This study aimed to investigate whether Ea_dyn_ can predict supine-to-prone hypotension. *Materials and Methods*: In this prospective, observational study, 47 patients who underwent elective spine surgery in the prone position were enrolled. Supine-to-prone hypotension is defined as a decrease in Mean Arterial Pressure (MAP) by more than 20% in the prone position compared to the supine position. Hemodynamic parameters, including systolic blood pressure (SAP), diastolic blood pressure, MAP, stroke volume variation (SVV), pulse pressure variation (PPV), stroke volume index, cardiac index, dP/dt, and hypotension prediction index (HPI), were collected in the supine and prone positions. Supine-to-prone hypotension was also assessed using two different definitions: MAP_prone_ < 65 mmHg and SAP_prone_ < 100 mmHg. Hemodynamic parameters were analyzed to determine the predictability of supine-to-prone hypotension. *Results*: Supine-to-prone hypotension occurred in 13 (27.7%) patients. Ea_dyn_ did not predict supine-to-prone hypotension [Area under the curve (AUC), 0.569; *p* = 0.440]. SAP_supine_ > 139 mmHg (AUC, 0.760; *p* = 0.003) and dP/dt_supine_ > 981 mmHg/s (AUC, 0.765; *p* = 0.002) predicted supine-to-prone hypotension. MAP_supine_, SAP_supine_, PPV_supine_, and HPI_supine_ predicted MAP_prone_ <65 mm Hg. MAP_supine_, SAP_supine_, SVV_supine_, PPV_supine_, and HPI_supine_ predicted SAP_prone_ < 100 mm Hg. *Conclusions*: Dynamic arterial elastance did not predict supine-to-prone hypotension in patients undergoing spine surgery. Systolic arterial pressure > 139 mmHg and dP/dt > 981 mmHg/s in the supine position were predictors for supine-to-prone hypotension. When different definitions were employed (mean arterial pressure < 65 mmHg in the prone position or systolic arterial pressure < 100 mmHg in the prone position), low blood pressures in the supine position were related to supine-to-prone hypotension.

## 1. Introduction

Supine-to-prone hypotension can occur after a positional change from supine to prone during general anesthesia [1,2]. Supine-to-prone hypotension is known to be caused by a decrease in venous return due to compression of the inferior vena cava and an increase in intrathoracic pressure [2,3]. The incidence of supine-to-prone hypotension is reported to be approximately 3% in awake patients [3], and 8.9–60% in anesthetized patients [4,5]. Intraoperative hypotension may contribute to increased morbidity, including acute kidney injury [6,7,8], myocardial injury [7], and stroke [9]. Furthermore, in the case of spine surgery performed in the prone position, hypotension can lead to visual loss and cord ischemia [4,10,11,12,13]. The risk of morbidity from hypotension increases with its severity and prolonged exposure to hypotension [7,8]. Therefore, it is important to predict and prevent hypotension in spine surgery [4].

Whether fluid therapy raises blood pressure depends on arterial tone, or, in other words, arterial elastance. Arterial elastance can be described as the ratio of blood pressure change to blood volume change [14]. Dynamic arterial elastance (Ea_dyn_) is a parameter that indicates arterial pressure responsiveness to fluid therapy using dynamic changes in pulse pressure and stroke volume under mechanical ventilation. Ea_dyn_ is defined as a ratio of pulse pressure variation (PPV) to stroke volume variation (SVV) [15]. In a previous study, Ea_dyn_ > 0.89 discriminated between fluid responders and non-responders in hypotensive patients [14]. Not only can Ea_dyn_ predict intraoperative hypotension, but it can also be useful for effectively treating hypotension [16]. However, it is not yet known whether Ea_dyn_ predicts supine-to-prone hypotension.

As the main mechanism of supine-to-prone hypotension is believed to be related to a decrease in venous return, patients with preoperative hypovolemia are considered susceptible to supine-to-prone hypotension [2,3]. However, it is unclear whether there is a relationship between volume status in the supine position and supine-to-prone hypotension. In this study, we investigated the predictability of Ea_dyn_ for supine-to-prone hypotension during elective spine surgery. Furthermore, we investigated whether other hemodynamic parameters could predict supine-to-prone hypotension. We also examined the predictability of hemodynamic parameters for supine-to-prone hypotension using different definitions of hypotension.

## 2. Materials and Methods

### 2.1. Ethics

This was a prospective, double-blind, observational study. This study was approved by the Ethics Board of the Kangbuk Samsung Hospital Institutional Review Board, Seoul, Korea (approval number: KBSMC 2021-04-038, approval date: 26 April 2021). Before patient enrollment, this study was registered at https://clinicaltrials.gov/study/NCT04850092 (accessed on 19 November 2023) (NCT04850092). This study was initiated after obtaining informed consent from all participants. This study was conducted at a single tertiary hospital in accordance with the principles of the Declaration of Helsinki.

### 2.2. Inclusion and Exclusion Criteria

The inclusion criteria were adult patients with American Society of Anesthesiologists Physical Status I–III, aged > 19 years, scheduled for elective spine surgery in the prone position, and scheduled for arterial cannulation. The exclusion criteria were as follows: arrhythmia, pulmonary disease, increased intracranial pressure, ejection fraction < 50% on preoperative echocardiography, signs of right heart failure, or obesity (body mass index > 40 kg/m^2^).

### 2.3. Anesthesia and Study Method

Premedication was not administered to any of the patients. All patients were catheterized with an 18-gauge angiocath into the vein in the forearm before entering the operating room. The fluid was connected and maintained at a rate of 40 cc/h. On arrival in the operating room, standard monitoring was initiated, including noninvasive blood pressure, electrocardiography, pulse oximetry, and anesthetic depth monitoring (Sedline^®^, Masimo Corp., Irvine, CA, USA). A neuromuscular monitoring device (TwitchViewTM; LTR Medical, Brisbane, Australia) was used to monitor the depth of the neuromuscular block. After checking the patency of the intravenous line, total intravenous anesthesia (TIVA) was initiated using a target-controlled infusion. For TIVA, 2% propofol was used as a Marsh model, and remifentanil was used as a Minto model. After 3 min of preoxygenation with 100% oxygen, anesthesia was induced using propofol at a target concentration of 4 mg/mL and remifentanil at a target concentration of 4 ng/mL. Once loss of consciousness was achieved, intravenous rocuronium 0.8 mg/kg was administered. After the train-of-four count reached 0, the airway was secured with an endotracheal tube, and mechanical ventilation was started. Mechanical ventilation was maintained with 50% air and oxygen. The tidal volume was set to 0.6–0.8 mL/kg, and the respiratory rate was adjusted to target an end-tidal carbon dioxide between 30 and 35 mmHg. Arterial catheterization was performed using a 20-gauge angiocath of the radial artery. Additional venous catheterization with an 18-gauge angiocath was performed in the forearm contralateral to the original venous line for intraoperative fluid therapy. The fluid flow rate was maintained at 1 mL/kg/h for each venous line.

### 2.4. Hemodynamic Data Collection

Hemodynamic data were retrieved from a cardiovascular monitoring device (HemoSphere, Edwards Lifesciences, Irvine, CA, USA) after connecting it to the radial arterial line using an Acumen IQ sensor (Edwards Lifesciences, Irvine, CA, USA). The hemodynamic data included Ea_dyn_, systolic blood pressure (SAP), diastolic blood pressure (DAP), mean arterial pressure (MAP), SVV, PPV, stroke volume index (SVI), cardiac index (CI), dP/dt, and hypotension prediction index (HPI). The device automatically calculates Ea_dyn_ by dividing the PPV by the SVV. HPI is an index ranging from 0 to 100, with higher scores predicting hypotension within 5 min [17].

The patients were left untouched in the supine position for several minutes before turning to the prone position. Once hemodynamic variables were stabilized, hemodynamic data (Ea_dyn_, SAP, DAP, MAP, SVV, PPV, SVI, CI, dP/dt, and HPI) in the supine position were recorded. Before turning the patients over, all monitors and breathing circuits were detached. After the participants turned from the supine position to the prone position, the monitoring equipment was reattached as soon as possible. Arterial and venous lines were attached during a position change. All participants were laid on Wilson’s frame, and abdominal pressure was avoided. After transitioning to the prone position, a timer was initiated. Five minutes later, the HemoSphere screen was captured, and the obtained image was utilized to record hemodynamic variables, including Ea_dyn_, SAP, DAP, MAP, SVV, PPV, SVI, CI, dP/dt, and HPI.

### 2.5. Definition of Supin-to-Prone Hypotension

Supine-to-prone hypotension was defined when MAP_prone_ after 5 min of position change decreased by >20% from MAP_supine_ [4]. Supine-to-prone hypotension was treated with fluid 300 mL (SVV > 13), ephedrine 4 mg (dP/dt < 400 mmHg/s), or phenylephrine 50 μg (SVR < 800 dynes/s/cm^−5^) [18]. Supine-to-prone hypotension was also assessed using different definitions: MAP_prone_ < 65 mmHg [6,8] and SAP_prone_ < 100 mmHg [19].

### 2.6. Statistical Analysis

This study aimed to determine whether Ea_dyn_ can predict a supine-to-prone condition with an area under the receiver operating characteristic (ROC) curve (AUC) of ≥0.8. In our pilot study, the incidence of supine-to-prone hypotension was 27% (unpublished data). Therefore, a sample size of 44 was calculated to test an AUC ≥ 0.8, with a power of 80% and an alpha of 0.05, considering a dropout rate of 20%.

Data are presented as mean (SD), median (interquartile range), and number (%). Mean, median, percentage difference, and 95% confidence intervals (CIs) were presented as necessary. Data were compared between the no-hypotension and hypotension groups. Continuous variables were tested for normal distribution using the Kolmogorov–Smirnov test. Student’s *t*-test was used to compare normally distributed variables. The Mann–Whitney U test was used for non-normally distributed and ordinal variables. For categorical variables, Pearson’s chi-square test or Fisher’s exact test were used, as appropriate.

A ROC curve was plotted to justify the predictability of hemodynamic variables for supine-to-prone hypotension. The ROC curve was analyzed for the threshold after assessment with the Youden index. The kappa index was calculated to evaluate the concordance of the three different definitions of supine-prone hypotension, and the strength of agreement was evaluated according to the Landis and Koch classification [20]. Data were analyzed using MedCalc^®^ Statistical Software version 20.014 (MedCalc Software Ltd., Ostend, Belgium; https://www.medcalc.org (accessed on 2 October 2023); 2021) and SPSS Statistics for Windows, Version 24.0 (IBM Corp., Armonk, New York, NY, USA). Statistical significance was set at *p* = 0.05.

## 3. Results

### 3.1. Patient Characteristics

**Figure 1:**
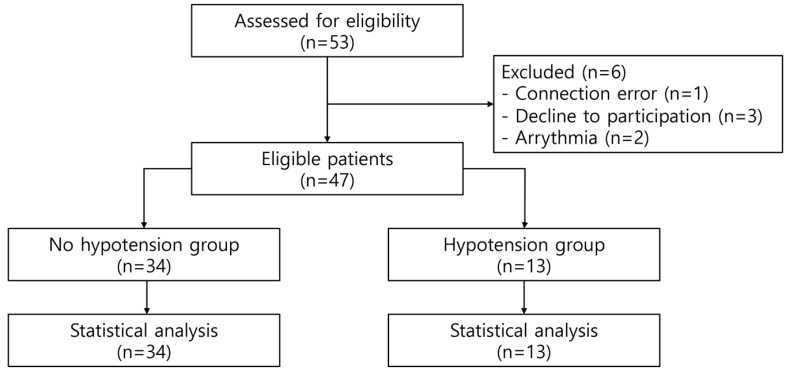
A flow diagram of the present study.

Data were collected from December 2021 to August 2022. After assessing the eligibility of the 53 patients, six of them were excluded for the following reasons: connection error (*n* = 1), decline to participate (*n* = 3), and arrhythmia (*n* = 2). Therefore, 47 patients were eligible to participate in this study. Supine-to-prone hypotension occurred in 13 (27.7%) patients. Furthermore, 34 patients (72.3%) did not have supine-to-prone hypotension (Figure 1). There were no differences in baseline characteristics between the no-hypotension and hypotension groups (Table 1).

### 3.2. Hemodynamic Variables

MAP_supine_ was higher [95 (22) mmHg] in the hypotension group than in the no hypotension group [81 (16) mmHg, *p* = 0.047], with a mean difference of −14 (95% CI, −29–0). SAP_supine_ was 142 (30) mmHg in the hypotension group and 113 (24) mmHg in the no hypotension group (mean difference, 9; 95% CI, −9–27; *p* = 0.002). Ea_dyn_ in the supine position did not differ between the two groups (*p* = 0.466). dP/dt was higher in the hypotension group [1140 (338) mmHg/s] than in the no hypotension group [828 (332) mmHg/s; mean difference, 312; 95% CI, −538 to −86, *p* = 0.008]. HPI_supine_ did not differ between the groups (*p* = 0.069; Table 2).

Hemodynamic and ventilatory parameters when MAP_prone_ < 65 mmHg was used as a definition of supine-to-prone hypotension are shown in Supplementary Table S1. MAP_supine_ was lower [67 (11) mmHg] in the hypotension group than in the no hypotension group [89 (18) mmHg; mean difference, 22; 95% CI, 10–35; *p* = 0.001]. Ea_dyn,supine_ was 1.2 (0.9–1.4) in the no hypotension group, while it was 1.2 (1.1–1.5) in the hypotension group, showing no differences between the two groups (*p* = 0.369). HPI_supine_ was significantly higher in the hypotension group [21 (5–54)] than in the no hypotension group [95 (61–98), *p* = 0.002).

Hemodynamic and ventilatory parameters when SAP_prone_ < 100 mmHg were used for the definition of supine-to-prone hypotension are presented in Supplementary Table S2. MAP_supine_ was lower [75 (20) mmHg] in the hypotension group than in the no hypotension group [92 (1) mmHg; mean difference, 17; 95% CI, 7–27; *p* = 0.002]. SVV_supine_ (*p* = 0.033) and PPV_supine_ (*p* = 0.013) were higher in the hypotension group than in the nonhypotension group. HPI_supine_ was higher in the hypotension group [96 (29–100)] than in the no hypotension group [18 (5–35), *p* = 0.001]. The airway pressure_prone_ was higher in the hypotension group than in the no hypotension group [mean difference, −3; 95% CI, −5–0; *p* = 0.038].

**Figure 2:**
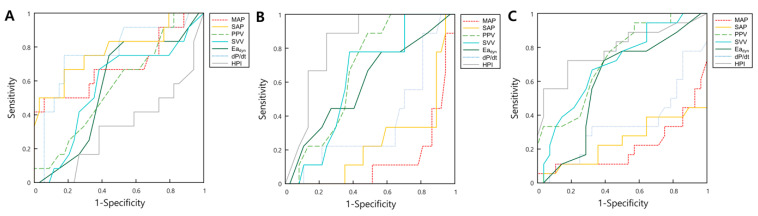
The receiver operator characteristic curves for hemodynamic parameters including MAP, SAP, PPV, SVV, Ea_dyn_ dP/dt, and HPI predict supine-to-prone hypotension using three different definitions of hypotension: Definition 1: MAP decrease > 20% compared to the supine position (**A**); Definition 2: MAP_prone_ < 65 mmHg (**B**); and Definition 3: SAP_prone_ < 100 mmHg (**C**). Abbreviations: MAP, mean blood pressure; SAP, systolic blood pressure; PPV, pulse pressure variation; SVV, stroke volume variation; Ea_dyn_, dynamic arterial elastance; HPI, hypotension prediction index.

The ROC curves are shown in Figure 2A. AUC, threshold, sensitivity, and specificity are shown in Table 3. Ea_dyn_ did not predict supine-to-prone hypotension (AUC, 0.569; *p* = 0.440). SAP_supine_ predicted supine-to-prone hypotension at a threshold of >139 (AUC, 0.760; 95% CI, 0.613–0.873, *p* = 0.003), with a sensitivity of 61.5% and a specificity of 82.4%. The AUC of dP/dt_supine_ for the prediction of supine-to-prone hypotension was 0.765 (95% CI, 0.617–0.877, *p* = 0.002).

ROC curves when MAP_prone_ < 65 mmHg was used as a definition of supine-to-prone hypotension are illustrated in Figure 2B. The AUCs (95% CI) of hemodynamic parameters for predicting supine-to-prone hypotension (MAP_prone_ < 65 mmHg) were MAP_supine_, 0.867 (0.736–0.948); SAP_supine_, 0.757 (0.610–0.870); PPV_supine_, 0.680 (0.528–0.808); and HPI_supine_, 0.832 (0.692–0.926; Table 3).

Figure 2C shows the ROC curves of hemodynamic parameters when SAP_prone_ < 100 mmHg was used as the definition of supine-to-prone hypotension. The AUCs (95% CI) of the hemodynamic parameters were as follows: MAP_supine_, 0.783 (0.639–0.890); SAP_supine_, 0.746 (0.598–0.862); SVV_supine_, 0.684 (0.532–0.812); PPV_supine_, 0.714 (0.564–0.836); and HPI_supine_, 0.790 (0.644–0.896) (Table 3).

Supplementary Table S3 shows the concordance between three different definitions of supine-to-prone hypotension: Definition 1, MAP decrease > 20% compared to the supine position; Definition 2, MAP_prone_ < 65 mmHg; and Definition 3, SAP_prone_ < 100 mmHg. There was a fair agreement between Definition 1 and Definition 2 (kappa index, 0.295) and Definition 2 × Definition 3 (kappa index, 0.349). The strength of agreement between Definitions 1 and 3 (kappa index, 0.517) was moderate.

## 4. Discussion

This study aimed to investigate whether Ea_dyn_ could predict supine-to-prone hypotension during elective spine surgery. However, Ea_dyn_ did not predict supine-to-prone hypotension, regardless of the applied definition. Among the hemodynamic parameters collected, SAP_supine_ > 139 mmHg and dP/dt > 981 mmHg/s predicted supine-to-prone hypotension (MAP decreased by >20% from MAP_supine_). Supine-to-prone hypotension was defined as MAP_supine_ < 65 mmHg, MAP_supine_ ≤ 75 mmHg, SAP_supine_ ≤ 97 mmHg, PPV > 16, and HPI > 51. When SAP_prone_ < 100 mmHg was used to define supine-to-prone hypotension, MAP_supine_ < 100 mmHg, MAP_supine_ ≤ 75 mmHg, SAP_supine_ ≤ 97 mmHg, SVV > 15, PPV > 13, and HPI > 31 predicted supine-to-prone hypotension.

In the prone position, an increase in intraabdominal and intrathoracic pressure causes a decrease in venous return, stroke volume, and arterial blood pressure [5,21]. Moreover, there are many things to care for while turning the patient into a prone position, including not only the vital signs but also the airways, monitoring sensors and devices, and pressure site management [22]. These might delay the diagnosis and treatment of supine-to-prone hypotension. As even a short exposure time to hypotension can exacerbate postoperative outcomes, it is crucial to predict and prevent supine-to-prone hypotension [4].

The risk factors for supine-to-prone hypotension were evaluated in previous studies [4]. TIVA using propofol and remifentanil increased the risk of supine-to-prone hypotension compared with inhalation anesthesia [1,23]. High remifentanil effect-site concentration, preoperative use of beta-blockers, and high MAP_supine_ were reported as risk factors for supine-to-prone hypotension [4]. Similarly, in our study, high SAP_supine_ (>139 mmHg) and dP/dt > 981 mmHg/s predicted supine-to-prone hypotension. This can be explained by activation of the baroreceptor reflex triggered by high blood pressure, which causes vasodilation and bradycardia after a position change [4]. However, because TIVA was performed in all patients, our study could not investigate the effect of propofol or remifentanil on supine-to-prone hypotension.

Our results showed that supine-to-prone hypotension patients had higher MAP_supine_ (95 mmHg) and higher dP/dt_supine_ (1140 mmHg/s).

A previous study suggested that fluid loading in the supine position before position change could prevent supine-to-prone hypotension [24]. From a similar point of view, we expected that fluid responsiveness would predict supine-to-prone hypotension. However, in the present study, Ea_dyn_ did not predict supine-to-prone hypotension. Furthermore, other parameters that represent volume responsiveness or status, such as SVV, PPV, and SVI, did not predict supine-to-prone hypotension. This corresponds to the previous study in that PPV did not show predictability for supine-to-prone hypotension [4]. We hypothesize that, although hypovolemia is a risk for supine-to-prone hypotension, intrathoracic pressure has a greater impact on the development of supine-to-prone hypotension.

Although there is no universal definition for supine-to-prone hypotension, the relative threshold of MAP decreased by >20% from MAP_supine_, which is generally used to define supine-to-prone hypotension in previous studies [4,5]. However, characterizing intraoperative hypotension by relative thresholds was suggested to be no better than using absolute thresholds in relation to postoperative complications [7]. The risk of poor major postoperative outcomes (acute kidney injury and myocardial injury) increases with a MAP < 65 mmHg. Furthermore, it has been reported that cord ischemia and visual loss in spine surgery are caused by MAP 50–70 mmHg [11,12]. Therefore, we also analyzed predictors of supine-to-prone hypotension using absolute thresholds (MAP_prone_ < 65 mmHg and SAP_prone_ < 100 mmHg). The concordance among the three definitions of supine-to-prone hypotension was quite consistent, but the predictors of supine-to-prone hypotension differed depending on which definition was used. In particular, as shown in Table 3, unlike high SAP_supine_ (>139 mmHg), which predicted supine-to-prone hypotension using a relative threshold, low SAP_supine_ (≤97 mmHg) predicted supine-to-prone hypotension using an absolute threshold. Also, when using relative threshold, a hyperdynamic state with high MAP and high dP/dt in the supine position predicted supine-to-prone hypotension (Table 2). On the other hand, when using the absolute definition, MAP_supine_ was low, and there was volume responsiveness (high pulse pressure variation), indicating an already hypotensive tendency (Table 3). Moreover, as described in the Supplementary Table S2, Ea_dyn_ (1.3) in the supine position was higher in the hypotension group, and the HPI was higher at 96 compared to 18 in the no hypotension group. Furthermore, MAP_prone_ was lower [64 (14) mmHg vs. 54 (9) mmHg] when applying MAP_prone_ < 65 mmHg than when applying MAP, which decreased by >20% from MAP_supine_ as a definition for supine-to-prone hypotension. Therefore, we believe that the risk of poor postoperative complications is more related to MAP_prone_ < 65 mmHg than to MAP decreased by >20% from MAP_supine_. The purpose of preventing intraoperative hypotension was to reduce the incidence of postoperative complications. Therefore, more research is needed to determine which definition of supine-to-prone hypotension is favorable in relation to postoperative outcomes in spine surgery.

There are some limitations to our study. First, this study was conducted using TIVA on all patients. We used TIVA in our study to avoid the effect of intraoperative neuromonitoring, which is performed frequently in our hospital. The hemodynamic changes in TIVA are different from those in inhalation anesthesia. TIVA lowers blood pressure and heart rate more than inhalation anesthesia [1,23]. Therefore, different results may be obtained if inhalation anesthesia is used. Second, because the hemodynamic variables were derived by the hemodynamic monitoring machine (HemoSphere, Edwards Lifesciences, Irvine, CA, USA), values such as stroke volume index and cardiac index are underestimated. However, these devices prioritize tracking trends over accuracy. Therefore, caution is advised when placing reliance on the absolute values of stroke volume and cardiac index recorded by the HemoSphere device in our research findings. Thirdly, we could not show the difference in the incidence of complications due to supine-to-prone hypotension after surgery between the two groups. Unfortunately, there were no occurrences of postoperative complications in either group, possibly due to the small sample size. Therefore, it would be valuable for future research with a larger sample size to investigate the difference in complications arising from supine-to-hypotension after surgery.

## 5. Conclusions

In conclusion, Ea_dyn_ did not predict supine-to-prone hypotension in patients undergoing spine surgery under general anesthesia using total intravenous anesthesia. Systolic arterial pressure greater than 139 mmHg and dP/dt greater than 981 mmHg/s in the supine position predicted supine-to-prone hypotension. Low blood pressure in the supine position was related to supine-to-prone hypotension, defined by absolute thresholds: mean arterial pressure below 65 mmHg in the prone position and systolic arterial pressure in the prone position below 100 mmHg.

## Figures and Tables

**Table 1:** Baseline demographics of patients.

	All Patients (*n* = 47)	No Hypotension (*n* = 34)	Hypotension (*n* = 13)	*p*-Value
Age, years	65 (12)	65 (13)	69 (9)	0.289
Sex, male/female	18/29 (38.3%/61.7%)	15/19 (44.1%/55.9%)	3/10 (23.1%/76.9%)	0.315
Height, cm	158 (8)	160 (8)	154 (7)	0.014
Body weight, kg	67 (13)	69 (13)	61 (12)	0.054
Body mass index, kg/m^2^	27 (5)	27 (5)	26 (4)	0.377
ASA PS	2 (2–3)	2 (2–3)	2 (2–3)	0.499
Smoker	6 (12.8%)	4 (11.8%)	2 (15.4%)	>0.999
Hypertension	29 (61.7%(	19 (55.9%)	10 (76.9%)	0.315
Alpha 2 receptor blocker	17 (36.2%)	10 (29.4%)	7 (53.8%)	0.119
Beta-blocker	5 (10.6%)	4 (11.8%)	1 (7.7%)	1.000
Calcium channel blocker	18 (38.3%)	12 (35.3%)	6 (46.2%)	0.493
Diabetes mellitus	11 (23.4%)	9 (26.5%)	2 (15.4%)	0.702
Dyslipidemia	18 (38.3%)	14 (41.2%)	4 (30.8%)	0.739

ASA PS, American Society of Anesthesiologists Physical Status. Data are presented as mean (SD), median (interquartile range), or number (%).

**Table 2:** A comparison of the hemodynamic and ventilatory parameters between the two groups in the supine and prone positions when MAP decreased by >20% from MAP_supine_ was used for the definition of supine-to-prone hypotension.

	No Hypotension (*n* = 34)	Hypotension (*n* = 13)	Difference (95% CI)	*p*-Value
Mean arterial pressure, mmHg				
Supine	81 (16)	95 (22)	−14 (−29–0)	0.047 *
Prone	84 (15)	64 (14)	20 (10–29)	<0.001 *
Systolic blood pressure, mmHg				
Supine	113 (24)	142 (30)	9 (−9–27)	0.002 *
Prone	112 (23)	88 (33)	24 (7–41)	0.007 *
Diastolic blood pressure, mmHg				
Supine	62 (12)	70 (16)	−8 (−17–1)	0.078
Prone	69 (14)	54 (16)	15 (6–25)	0.003 *
Stroke volume index, mL/m^2^				
Supine	29 (7)	29 (10)	−1 (−6–5)	0.846
Prone	27 (7)	28 (7)	−2 (−6–3)	0.484
Cardiac index, L/min/m^2^				
Supine	2.1 (0.6)	2.5 (0.9)	−0.4 (−0.8–0)	0.179
Prone	1.8 (0.5)	2.0 (0.5)	−0.2 (−0.5–0)	0.210
Stroke volume variation, %				
Supine	14.5 (12.0–18.0)	16.0 (11.5–18.3)	1.0 (−3.0–4.0)	0.625
Prone	14.0 (10.0–17.0)	15.0 (9.8–18.5)	0 (−3.0–5.0)	0.849
Pulse pressure variance, %				
Supine	17.0 (12.0–22.0)	17.0 (13.8–22.3)	1.0 (−3.0–6.0)	0.592
Prone	13.0 (11.0–18.0)	14.0 (9.8–22.8)	1.0 (−4.0–6.0)	0.567
Ea_dyn_				
Supine	1.1 (0.9–1.5)	1.2 (1.2–1.3)	0.1 (−0.1–0.3)	0.466
Prone	1.1 (0.8–1.3)	1.1 (0.9–1.3)	0.0 (−0.2–0.2)	0.886
dP/dt, mmHg/s				
Supine	828 (332)	1140 (338)	−312 (−538 to −86)	0.008 *
Prone	651 (243)	610 (258)	41 (−126–208)	0.623
Hypotension prediction index				
Supine	33.0 (14.0–96.0)	13.0 (2.5–60.0)	−13.0 (−36.0–1.0)	0.069
Prone	25.0 (11.0–68.0)	94.5 (49.5–100)	41.0 (1.0–78.0)	0.021 *
Tidal volume, mL	443 (63)	434 (62)	10 (−32–51)	0.639
Airway pressure, mmHg				
Supine	17 (3)	16 (3)	1 (−2–3)	0.595
Prone	19 (5)	16 (4)	3 (0–5)	0.074
Δ (prone-supine)	2.0 (0–3.0)	0 (−0.25–1.25)	−1.0 (−3.0–0)	0.051

* *p* < 0.05. Ea_dyn_, dynamic arterial elastance; CI, confidence interval. Data are presented as mean (SD) or median (interquartile range).

**Table 3:** The area under the curve, threshold, sensitivity, and specificity of hemodynamic variables to predict supine-to-prone hypotension, using three different definitions.

	AUC (95% CI)	Threshold	Sensitivity (%)	Specificity (%)	*p*-Value
Definition 1: Mean arterial pressure decrease > 20% compared to the supine position					
Mean arterial pressure	0.678 (0.525–0.806)	>103	46.2	94.1	0.076
Systolic blood pressure	0.760 (0.613–0.873)	>139	61.5	82.4	0.003 *
Stroke volume index	0.517 (0.370–0.662)	>30	50.0	71.4	0.869
Pulse pressure variance	0.551 (0.399–0.696)	>9	100.0	17.7	0.580
Ea_dyn_	0.569 (0.416–0.713)	>1.1	76.9	55.9	0.440
dP/dt	0.765 (0.617–0.877)	>981	75.0	82.4	0.002 *
Hypotension prediction index	0.678 (0.524–0.808)	≤11	50.0	82.4	0.055
Definition 2: Mean arterial pressure < 65 mmHg in the prone position					
Mean arterial pressure	0.867 (0.736–0.948)	≤75	88.9	79.0	<0.001 *
Systolic blood pressure	0.757 (0.610–0.870)	≤97	66.7	89.5	0.004 *
Stroke volume variation	0.632 (0.478–0.768)	>15	77.8	63.2	0.144
Pulse pressure variance	0.680 (0.528–0.808)	>16	88.9	52.6	0.022 *
Ea_dyn_	0.596 (0.443–0.737)	>1	77.8	42.1	0.381
dP/dt	0.631 (0.476–0.768)	≤815	77.8	64.9	0.221
Hypotension prediction index	0.832 (0.692–0.926)	>51	88.9	75.7	<0.001 *
Definition 3: Systolic blood pressure < 100 mmHg in the prone position					
Mean arterial pressure	0.783 (0.639–0.890)	≤75	63.16	85.71	<0.001 *
Systolic blood pressure	0.746 (0.598–0.862)	≤97	52.63	100	0.002 *
Stroke volume variation	0.684 (0.532–0.812)	>15	63.16	67.86	0.020 *
Pulse pressure variance	0.714 (0.564–0.836)	>13	94.74	42.86	0.005 *
Ea_dyn_	0.601 (0.447–0.741)	>1.1	73.68	60.71	0.242
dP/dt	0.653 (0.498–0.787)	≤815	66.67	71.43	0.088
Hypotension prediction index	0.790 (0.644–0.896)	>35	72.22	82.14	<0.001 *

* *p* < 0.05. Ea_dyn_, dynamic arterial elastance; CI, confidence interval.

## Data Availability

The data used to support the findings of this study are available from the corresponding author upon reasonable request.

## Supplementary Materials

The following supporting information can be downloaded: https://www.mdpi.com/article/10.3390/medicina59122049/s1, Table S1: Hemodynamic and ventilatory parameters when mean blood pressure < 65 mmHg were used to define supine-to-prone hypotension. Table S2: Hemodynamic and ventilatory parameters when systolic blood pressure < 100 mmHg were used to define supine-to-prone hypotension. Table S3: Concordance between the three definitions of supine-to-prone hypotension.
