# Supplementary material for: Dynamic Arterial Elastance as a Predictor of Supine-to-Prone Hypotension (SuProne Study): An Observational Study
Source: Medicina (Kaunas). 2023 Nov 21;59(12):2049. doi: 10.3390/medicina59122049 (PMC10744433; doi:10.3390/medicina59122049)
Supplement: Supplementary file 1 [file medicina-59-02049-s001.zip › medicina-2706493-supplementary.pdf]

Supplementary Table S1. Hemodynamic and ventilatory parameters when mean blood pressure <65 mmHg were used to define supine-to-prone hypotension.

	No hypotension (n = 38)	Hypotension (n = 9)	Difference (95% CI)	*P*-value
Mean arterial pressure, mmHg				
Supine	89 ± 18	67 ± 11	22 (10–35)	0.001*
Prone	84 ± 13	54 ± 9	30 (20–39)	<0.0001*
Systolic blood pressure, mmHg				
Supine	125 ± 29	102 ± 22	23 (3–44)	0.028*
Prone	112 ± 26	77 ± 15	35 (17–54)	<0.0001*
Diastolic blood pressure, mmHg				
Supine	68 ± 13	50 ± 8	18 (9–27)	<0.0001*
Prone	70 ± 13	43 ± 7	26 (20–32)	<0.0001*
Stroke volume index, mL/m^2				
Supine	29 ± 8	27 ± 8	2 (-3–8)	0.399
Prone	27 ± 7	25 ± 7	2 (-3–8)	0.399
Cardiac index, L/min/m^2				
Supine	2.2 ± 0.7	2.2 ± 0.7	0 (-0.5–0.6)	0.891
Prone	1.8 ± 0.5	1.9 ± 0.6	-0.1 (-0.4–0.3)	0.757
Stroke volume variation, %				
Supine	15(10–18)	17 (15–18)	2.0 (-1.0–6.0)	0.223
Prone	12 (10–16)	19 (15–26)	7.0 (3–13)	0.007*
Pulse pressure variance, %				
Supine	16 (12–22)	18 (17–23)	3.5 (-1.0–8.0)	0.096

Prone	13 (10–16)	21 (13–37)	7 (0–19)	0.028*
Ea_{dyn}				
Supine	1.2 (0.9–1.4)	1.2 (1.1–1.5)	0.1 (-0.1–1.5)	0.369
Prone	1.1 (0.9–1.3)	1.2 (0.8–1.3)	0 (-0.3–0.3)	0.881
dP/dt, mmHg/s				
Supine	936 ± 371	799 ± 288	137 (-130–405)	0.307
Prone	691 ± 237	432 ± 154	259 (91–427)	0.003*
Hypotension prediction index				
Supine	21 (5–54)	96 (61–98)	59 (24–82)	0.002*
Prone	25 (11–57)	100 (100–100)	75 (48–88)	<0.0001*
Tidal volume, mL	443 ± 63	434 ± 62	10 (-32–51)	0.639
Airway pressure, mmHg				
Supine	444 ± 64	427 ± 55	17(-30–64)	0.466
Prone	16 ± 3	17 ± 4	-1 (-4–1)	0.334
Δ (prone-supine)	18 ± 4	20 ± 5	-2 (-5–1)	0.246

**P* < 0.05

Ea_{dyn}, dynamic arterial elastance

Data are presented as mean ± SD or median (interquartile range).

Supplementary Table S2. Hemodynamic and ventilatory parameters when systolic blood pressure <100 mmHg were used to define supine-to-prone hypotension.

	No hypotension (n = 28)	Hypotension (n = 19)	Difference (95% CI)	*P*-value
Mean arterial pressure, mmHg				
Supine	92 ± 15	75 ± 20	17 (7–27)	0.002*
Prone	88 ± 13	63 ± 11	25 (17–32)	<0.0001*
Systolic blood pressure, mmHg				
Supine	130 ± 24	107 ± 31	23 (7–39)	0.006*
Prone	124 ± 16	79 ± 20	45 (35–55)	<0.0001*
Diastolic blood pressure, mmHg				
Supine	70 ± 11	56 ± 14	14 (6–21)	0.001*
Prone	74 ± 12	51 ± 10	23 (17–30)	<0.0001*
Stroke volume index, mL/m^2				
Supine	29 ± 8	28 ± 7	1 (-4–6)	0.603
Prone	28 ± 7	25 ± 7	3 (-1–7)	0.122
Cardiac index, L/min/m^2				
Supine	2.2 ± 0.7	2.3 ± 0.7	-0.2 (-0.6–0.3)	0.471
Prone	1.9 ± 0.5	1.8 ± 0.5	0.1 (-0.2–0.4)	0.627

Stroke volume variation, %				
Supine	13.5 (10–17)	17 (13.3-20)	3 (0–6)	0.033*
Prone	11.0 (9–14.5)	17 (15-24)	6 (4–9)	<0.001*
Pulse pressure variance, %				
Supine	15.5 (11–20.5)	18 (16.3–27)	5 (1–9)	0.013*
Prone	13 (9.5–14)	17 (13.3–24.5)	6 (2–10)	0.002*
Ea_{dyn}				
Supine	1.05 (0.90–1.50)	1.3 (1.125–1.40)	0.10 (-0.1–0.3)	0.243
Prone	1.10 (0.85–1.30)	1.1 (0.9–1.2)	0 (-0.20–0.2)	0.913
dP/dt, mmHg/s				
Supine	976 ± 337	805 ± 373	171 (-43–385)	0.114
Prone	757 ± 204	460 ± 189	297 (176–418)	<0.0001*
Hypotension prediction index				
Supine	18 (5–35)	96 (29–100)	50 (16–73)	0.001*
Prone	13 (9–31)	100 (68–100)	75 (51–86)	<0.0001*
Tidal volume, mL	433 ± 62	452 ± 62	-19 (-56–19)	0.316
Airway pressure, mmHg				
Supine	16 ± 3	17 ± 3	-1 (-3–0)	0.128
Prone	17 ± 3	20 ± 5	-3 (-5–0)	0.038*
Δ (prone-supine)	1.5 (0–3.0)	1.0 (0–4.5)	0 (-1.0–2.0)	0.726

*$P < 0.05$

Ea_{dyn}, dynamic arterial elastance

Data are presented as mean ± SD, or median (interquartile range).

Supplementary Table S3. Concordance between the three definitions of supine-to-prone hypotension

	Kappa index	*P*-value
Definition 1 * definition 2	0.295	0.037†
Definition 2 * definition 3	0.349	0.013†
Definition 1 * definition 3	0.517	<0.001†

†$P < 0.05$

Definition 1: mean arterial pressure decrease >20% compared to the supine position

Definition 2: mean arterial pressure <65 mmHg in the prone position

Definition 3: systolic blood pressure <100 mmHg in the prone position
